# Supplementary material for: Factors Affecting Continued Participation in Tuberculosis Contact Investigation in a Low-Income, High-Burden Setting
Source: Trop Med Infect Dis. 2020 Jul 29;5(3):124. doi: 10.3390/tropicalmed5030124 (PMC7558864; doi:10.3390/tropicalmed5030124)
Supplement: Supplementary file 1 [file tropicalmed-05-00124-s001.zip › supplementary files - zip/Supplementary Questionnaire S1 - KAP Questionnaire.pdf]

Supplementary Questionnaire S1: Knowledge, Attitude and Practice (KAP) Questionnaire

Health facility	
Date of interview	………../………../
Interviewer's full name	
Interviewee's full name	
Study number of interviewee	_ _ _ _ _ _ _
Did the contact agree to get involved in the survey?	☐Agree<br>☐Disagree<br>☐Can't contact

The study is being conducted by Infectious Disease Society Kota Kinabalu, Sabah in collaboration with Menzies School of Health Research and University Malaysia Sabah. You are invited to complete a questionnaire about knowledge, perception and practices of TB within close contacts of patients who are infected with TB. The purpose of this questionnaire is to find out your opinions about tuberculosis screening. We also want to learn about what factors might make it difficult for you to attend screening, and what can be done to improve your access to tuberculosis screening. This information will be used to help improve the way that the National Tuberculosis Program conducts screening of contacts of tuberculosis patients in the future. This will take about 10 minutes. Thank you for completing this survey!

I. BASIC INFORMATION

#	Question	Answer	Code
Q1.1	What is your relationship with <name of TB patient>?<br><br>*(Check one option)*	☐Father/mother<br>☐Spouse<br>☐Children<br>☐Siblings<br>☐Other relative<br>☐Other (specify……………………...)	1<br>2<br>3<br>4<br>5<br>88
Q 1.2	Gender	☐Male<br>☐Female	1<br>2
Q1.3	What is the highest level of education you have finished?<br>*(Check one option)*	☐Bachelors' and above<br>☐Diploma<br>☐Form 6/ A Levels/ Matriculation/ HSC Secondary<br>☐SPM/ O Levels<br>☐PMR/ SRP/ LSC<br>☐Primary 6<br>☐No formal education	1<br>2<br>3<br><br>4<br>5<br>6<br>7
Q1.4	Currently, which of the following best describes your current main job?<br><br>*(Check one option)*	☐Unemployed<br>☐Student<br>☐Government employee<br>☐Private employee<br>☐Self-employed<br>☐Housewife	1<br>2<br>3<br>4<br>5<br>6

		☐Other (specify……………………..)	88
Q1.5	What was the average income that you earn **each month** (including official and other sources)?	☐< RM710<br>☐RM 710 – RM 1000<br>☐RM 1001 – RM 2000<br>☐RM 2001 – RM3000<br>☐> RM3000	1<br>2<br>3<br>4<br>5
Q1.6	Race	☐Malay<br>☐Chinese<br>☐Indian<br>☐Bumiputera<br>☐Others (please state)………………..	1<br>2<br>3<br>4<br>88
Q1.7	Marital status	☐ Single<br>☐Married<br>☐ Widower<br>☐ Divorced	1<br>2<br>3<br>4
Q1.8	Number of household members	………………	

II. KNOWLEDGE OF HOUSEHOLD CONTACT

#	Question	Answer	Code		
Q2.1	I am going to read a list of possible symptoms. For each one, please tell me if you think it is **commonly** associated with TB? For each option, say either "yes", "no" or "I don't know".<br>*(Check one or more options)*	Cough	Yes<br>1☐	No<br>2☐	Don't know<br>99☐
		Fatigue	1☐	2☐	99☐
		Leg pain	1☐	2☐	99☐
		Weight loss	1☐	2☐	99☐
		Night sweats	1☐	2☐	99☐
		Increased appetite	1☐	2☐	99☐
		Cough up blood	1☐	2☐	99☐
		Chest pain	1☐	2☐	99☐
		Dizziness	1☐	2☐	99☐

		Fever	1☐	2☐	99☐
		Difficulty breathing	1☐	2☐	99☐

Q2.2	**Which of the following activities causes a person with TB to spread the infection to others? For each option, state "Yes", "No", or "Don't know"** *(Check one or more options)*	**Response**		
		**Yes**	**No**	**Don't know**
	When patient sneezes	☐1	☐2	☐99
	When patient talks	☐1	☐2	☐99
	When eating and drinking using the same utensils (such as bowl, utensils, glass) as the person with TB	☐1	☐2	☐99
	When sleeping in the same room as the person with TB	☐1	☐2	☐99
	When using same belongings (towels, clothes, napkins)	☐1	☐2	☐99
	When hugging or kissing the person with TB	☐1	☐2	☐99
	When using the same toilet	☐1	☐2	☐99
	When having sexual intercourse with the person with TB	☐1	☐2	☐99

Q2.3	**Now I am going to read some statements about natural history of TB. Some of them are true and some are not true. For each option, say if it is "true", "false" or you "Don't know".** *(Check one or more options)*			
	**Statement**	**Response**		
		**True**	**False**	**Don't know**
	TB is caused by an infection	☐1	☐2	☐99
	TB is caused by living in an unhygienic environment	☐1	☐2	☐99
	Tuberculosis is acquired by being inherited from your parents	☐1	☐2	☐99
	Some people have extrapulmonary TB which cannot be spread from person to person	☐1	☐2	☐99
	Smoking increases susceptibility to TB	☐1	☐2	☐99

	Children are at a greater risk of developing TB than adults	☐1	☐2	☐99
	People with HIV/AIDS have a higher risk of developing TB than a person without HIV	☐1	☐2	☐99
	Pregnant women are at a greater risk of developing TB than people who are not pregnant	☐1	☐2	☐99
	People with weakened immune system due to chronic disease such as diabetes are at a higher risk of developing TB	☐1	☐2	☐99
	People with poor nutrition have a higher risk of developing TB	☐1	☐2	☐99
	TB can be completely cured if a patient takes treatment.	☐1	☐2	☐99
	Traditional medicine can be used to cure tuberculosis	☐1	☐2	☐99

III. PERCEPTION OF HOUSEHOLD CONTACT TOWARD TB CONTACT INVESTIGATION

#	Question	Answer	Code	
Q3.1	Do you think that there is discrimination against people with TB?<br>*(Check one option)*	☐Yes<br>☐No<br>☐Don't know	1<br>2<br>88	
Q3.2	How would you rate your risk of developing TB compared to a member of the general population?<br>*(Check one option)*	☐Much lower risk<br>☐Lower risk<br>☐Similar risk<br>☐Higher risk<br>☐Much higher risk	1<br>2<br>3<br>4<br>5	
Q3.3	<u>For defaulters only</u>:<br>**State whether you agree or disagree with each of the following statements**<br>*(Check one or more options)*	**I did not attend my clinic appointment because:**<br><br>The distance to travel from my home to preventative district health center is too far	**Agree**<br><br>1 ☐	**Disagree**<br><br>2☐
		I worry about the harmful effect of an Xray	1 ☐	2☐
		I wanted to be examined in a private health clinic instead	1 ☐	2☐

		I am worried about discrimination from other people toward myself and my family	1 ☐	2☐
		The patient in my household recovered, so it is unnecessary to follow up the screening anymore	1 ☐	2☐
		I didn't remember the appointment date	1 ☐	2☐
		It is time-consuming. It was difficult to get time off work or study	1 ☐	2☐
		I was pregnant	1 ☐	2☐
		Others, please specify :		
3.4	I am going to read a statement, and I would like you to choose one option that reflects your opinion.<br>"Overall, the TB screening program is beneficial for me and my family"	☐Strongly disagree<br>☐Disagree<br>☐Neither agree nor disagree<br>☐Agree<br>☐Strongly agree	1<br>2<br>3<br>4<br>5	

IV. PRACTICE OF HOUSEHOLD CONTACTS IN TB CONTACT INVESTIGATION

#	Question	Answer	Code
Q 4.1	Who would you talk to about your illness if you had TB?	☐Doctor or other medical worker<br>☐Spouse<br>☐Parent<br>☐Child(ren)<br>☐Other family member<br>☐Close friend<br>☐No one<br>☐Other (please specify):<br>....................	1<br>2<br>3<br>4<br>5<br>6<br>7<br>88
Q 4.2	What would you do if you thought you had symptoms of TB?	☐ Go to health facility<br>☐ Go to pharmacy<br>☐ Go to traditional healer<br>☐ Pursue other self-treatment options (eg. Herbs,etc)<br>☐ Other (please specify):<br>....................	1<br>2<br>3<br>4<br><br>88
Q 4.3	If you had symptoms of TB, when would you go to the health facility?	☐ When treatment on my own does not work<br>☐ When symptoms that look like TB signs last for 3–4 weeks	1<br><br>2<br><br>3

		☐ As soon as I realize that my symptoms might be related to TB<br>☐ I would not go to the doctor	4

V. ACCESSIBILITY OF HOUSEHOLD CONTACTS TO TB CONTACT INVESTIGATION

#	Question	Answer	Code
Q 5.1	How did the health care worker inform you about your contact screening appointment?	☐Telephone<br>☐Visit to my house<br>☐Sent a letter to my house<br>☐They did not contact me to remind me<br>☐Other (specify……………………….)	1<br>2<br>3<br>4<br>88
Q 5.2	How far do you live from the District clinic?<br>(*Check the one answer that is most suitable)*	☐Less than 2 km from the Clinic<br>☐Between 2 km and 9km from the Clinic<br>☐From 10 km to 15 km<br>☐16km or above	1<br>2<br>3<br>4
Q 5.3	How would you usually get to the district health clinic?<br>(*Check the one answer that is most suitable)*	☐Walking<br>☐Car<br>☐Motorbike<br>☐Bus<br>☐Other (specify……………………….)	1<br>2<br>3<br>4<br>88
