# Supplementary material for: Factors Affecting Continued Participation in Tuberculosis Contact Investigation in a Low-Income, High-Burden Setting
Source: Trop Med Infect Dis. 2020 Jul 29;5(3):124. doi: 10.3390/tropicalmed5030124 (PMC7558864; doi:10.3390/tropicalmed5030124)
Supplement: Supplementary file 1 [file tropicalmed-05-00124-s001.zip › supplementary files - zip/Supplementary Table S1 - Accessibility to TB contact investigation.pdf]

Supplementary Table S1: Accessibility to TB contact investigation among participants

Characteristics (n=237)	Frequency (%)
**Information**	
Telephone	32 (13.5)
Visit to my house	9 (3.8)
Sent a letter to my house	160 (67.5)
Visit to school or workplace	26 (11.0)
Other (passive screening)	10 (4.2)
**Distance**	
Less than 2 km from the Clinic	3 (1.3)
Between 2 km and 9km from the Clinic	195 (82.3)
From 10 km to 15 km	39 (16.5)
16km or above	0 (0.0)
**Transportation** (how would you normally get to the clinic?')	
Walking	0 (0.0)
Car	167 (70.5)
Motorcycle	2 (0.8)
Bus	15 (6.3)
Other (charter transport)	53 (22.4)
